# Health literacy in individuals with knee pain—a mixed methods study

**DOI:** 10.1186/s12889-023-16585-9

**Published:** 2023-08-29

**Authors:** Charlotte Sylwander, Astrid Klopstad Wahl, Maria L.E Andersson, Emma Haglund, Ingrid Larsson

**Affiliations:** 1 School of Health and Welfare, Halmstad University, Halmstad, SE-30118 Sweden; 2 Spenshult Research and Development Centre, Halmstad, Sweden; 3 Department of Interdisciplinary Health Sciences, University of Oslo, Oslo, Norway; 4 Department of Clinical Sciences, Section of Rheumatology, Lund University, Lund, Sweden; 5 Department of Environmental and Biosciences School of Business, Innovation and Sustainability, Halmstad University, Halmstad, Sweden

**Keywords:** Health literacy, Health promotion, Knee pain, Chronic pain, Knee osteoarthritis, Patient perspective, Mixed methods study

## Abstract

**Background:**

Low health literacy is associated with worse pain and poorer self-management. This study (1) examined the level of health literacy and associations with lifestyle habits, health status, chronic pain, and radiographic knee osteoarthritis; and (2) explored experiences illuminating health literacy among individuals with knee pain.

**Methods:**

A convergent parallel mixed-methods design was used, including 221 individuals. Health literacy was assessed by HLS-EU-Q16 and eHEALS. The questionnaire included questions on lifestyle habits, health status, and pain distribution. Radiographic knee osteoarthritis was assessed with x-rays. Associations were analysed using logistic regression analyses. Individual semi-structured interviews were conducted (*n* = 19) and analysed with qualitative content analysis.

**Results:**

The result showed that 71% reported sufficient health literacy. Higher education, healthy lifestyle habits, better general health, and absence of widespread pain were associated with sufficient health literacy. Experiences regarding health literacy influencing the decision-making process toward a decision on action comprised: (1) searching for information actively or passively; (2) processing of the information included being informed, critical, and interpretive; and (3) taking a stand on the information based on trustfulness and motivation.

**Conclusion:**

Seven out of 10 reported sufficient health literacy. Despite this, unhealthy lifestyles were common, suggesting that having sufficient HL is not enough for a behavioural change and the decision-making process, including different phases such as searching, processing, and taking a stand on health information is important to consider. More research on health literacy is needed to gain knowledge of how best to develop health promotion in individuals with knee pain.

**Supplementary Information:**

The online version contains supplementary material available at 10.1186/s12889-023-16585-9.

## Introduction

Health literacy (HL) is a key factor in the 2030 agenda for sustainable development [1]. HL is an important factor in preventive work for various long-term diseases such as osteoarthritis and chronic pain [2]. HL is defined as “people’s knowledge and competence to access, understand, appraise, and apply health information in order to take decisions in everyday life concerning healthcare, disease prevention and health promotion to maintain or improve quality of life during the life course " [3]. In Europe, almost half of the adult population report low HL, and people of older ages, low education, low socioeconomic status [4], and immigrants are particularly affected [5]. A low level of HL is associated with poorer general health [6], a greater need for healthcare [4], and a higher cost to society [2]. Individuals with low HL also participate less in disease prevention and are less compliant with treatment plans [7].

HL and self-management have a strong association, and risk factors for noncommunicable disease development may go unnoticed for many individuals in their everyday life [8]. Due to the often-slow onset of symptoms, it can be challenging to comprehend that continuous unhealthy lifestyle decisions can lead to, for example, the development of knee osteoarthritis (KOA) or chronic pain. Knee pain can be an early sign of KOA and an increased risk of chronic widespread pain (CWP) [9, 10], which can cause impairments, activity limitations, and participation restrictions [11–14] and are common causes of sick leave [15, 16]. Self-management, such as physical activity and weight loss (if overweight), are among the recommendations for managing KOA and chronic pain [17, 18]. Patient education is often included in self-management interventions for chronic pain [19] but studies report that individuals struggle to take decisions in everyday life and following these recommendations [20, 21]. One of several reasons could be low HL.

A low level of HL has been reported in individuals with chronic pain [22]. It is associated with higher pain intensity, worse pain problems [23, 24], poorer self-management [22] and an increased risk of anxiety and depression [25]. Low HL could also increase the risk of analgesic misuse [24] and has been reported as an obstacle when implementing health-promoting interventions for individuals with KOA [26].

On the other hand, a higher level of HL is linked to better self-management and pain management [23]. After a short educational intervention, an increased level of self-management was found in individuals with KOA [27]. However, individuals with low HL did not respond as well to the same intervention [27].

Since KOA is one of the most common musculoskeletal diseases [28], and 25–35% of adults live with chronic pain [29], studying HL in a group with the onset symptom of knee pain is essential to gain knowledge of how best to facilitate health promotive work [2, 22]. There is a lack of knowledge about the association between HL [30], and few studies have examined HL in individuals with symptom onset. Therefore, it is important to explore HL in individuals with early symptoms of KOA and chronic pain from different perspectives to obtain a more comprehensive understanding.

The overall aim was to understand health literacy among individuals with knee pain, specifically (1) to study the level of health literacy and associations with lifestyle habits, health status, chronic pain, and radiographic KOA and (2) to explore experiences illuminating health literacy among individuals with knee pain.

## Methods

### Design and setting

A convergent parallel mixed-methods design was applied. The design aimed to obtain different but complementary data on the same phenomenon to understand the research problem from different perspectives [31]. The procedure of the convergent parallel design can be explained through four steps [31]. Step (1) separately but simultaneously development of design and data collection of the quantitative and qualitative data; (2) separate analysis; (3) merging of the two sets of results by identifying and comparing similarities and differences; (4) summarising and discussing the interpretation of the merged results, aiming for a more complete and comprehensive understanding of HL in individuals with knee pain. The current study had an interactive level of interaction, meaning that there was a direct interaction between the quantitative and qualitative methods of the study [31]. The direct interaction is the theoretical health literacy framework [32], of which the questionnaire and the interview questions are based on. The priority was the same for the quantitative and qualitative data, and the integration occurred after the quantitative and qualitative results with a merged result and discussion.

According to the definition of HL, *health* in HL represents both health in relation to disease (including health care and disease prevention) and health as health promotion and general health [3]. Therefore, the results are presented using a two-dimensional approach highlighting the group with sufficient HL and limited HL.

The study is a part of the Halland knee OA cohort study (HALLOA) [33], registered at ClinicalTrials.gov (NCT04928170), and followed The Good Reporting of A Mixed Methods Study (GRAMMS) [34].

### Participants

The HALLOA study includes individuals with knee pain, and the overall intention is to study the early disease process of radiographic KOA (rKOA) and CWP. The participants were recruited in the southwest of Sweden via primary health care clinics when searching for care for knee pain and advertisement in the local newspapers [33]. At baseline, 306 individuals were included, and the participants are followed up annually for five years. This study includes data from the mid-follow-up period 2–3 years after the baseline, which occurred during 2019–2022.

### Quantitative part

At the mid-follow-up, 251 participants participated (18% dropout rate), and of these, 221 had completed the health literacy questionnaires and were included in the present study (67% (148) were women, mean age 56 ± 8 years old). The 30 non-responders were younger than the responders (49 ± 10 years, *p* < 0.001) and had a higher level of education (*p* = 0.044). No other differences were found. Why former participants decided not to respond in the follow-up is not known.

### Qualitative part

A purposeful sample of 19 individuals (11 women, 8 men) among the 251 participants was selected to participate in the interviews. The purposeful sample was chosen to represent a variety of women and men, ages, sociodemographics, educational levels, and reported lifestyle factors (BMI, physical activity, sedentariness, diet, smoking, snuff use, and alcohol intake). Participants’ sociodemographic and clinical data are presented in Table 1.

**Table 1:** Sociodemographic and clinical data of the interviewed participants. Presented as numbers (n), unless otherwise stated

	All *n* =19	Sufficient HL *n* =9	Limited HL *n* =10
Women / men	11 / 8	6 / 3	5 / 5
Age in years, median (range)	52 (43–62)	47 (51–60)	56.5 (43–62)
Native-born / foreign-born	16 / 3	6 / 3	10 / 0
In a relation / living alone	16 / 3	7 / 2	9 / 1
Level of education, Compulsory school / secondary / university	5 / 8 / 6	0 / 5 / 4	5 / 3 / 2
BMI^a^, Normal / overweight / obese	6 / 7 / 6	4 / 3 / 2	2 / 4 / 4
Meets recommendations for physical activity^b^	10	5	5
Sedentary, hours median (range)	6 (1.5–11.5)	6 (3–11.5)	4.5 (1.5–9)
Pain group, NCP / CRP / CWP	1 / 10 / 8	0 / 5 / 4	1 / 5 / 4
KOA^c^	9	3	6

‘Sufficient HL’ was defined as having a sufficient level of general HL and electronic HL. ^a^Normal BMI =18.5–24.9 kg/m^2^; overweight =25.0–29.9; obesity ≥30.0.^b^WHO recommendations: 150–300 minutes of moderate intensity or 75–150 minutes of vigorous intensity/ week. ^c^Having a score ≥1 on the Ahlbäck scale for KOA

*BMI *Body mass index, *KOA *Knee osteoarthritis, *NCP *No chronic pain, *CRP *Chronic regional pain, *CWP *Chronic widespread pain

### Data collection

#### Quantitative data

Data pertaining to dependent variables were pulled from the Swedish translation of the Health Literacy Survey European Questionnaire short form (HLS-EU-Q16 SE) [35] and the eHealth Literacy Scale (Sw-eHEALS) [36]. The HLS-EU-Q16 SE includes 16 questions that measure an individual’s perception of their ability to find, understand, interpret, and apply health information, or general HL (GHL). The individual's answered how they perceived the difficulty of each question. There is a 4-point Likert-like scale with the answers: ‘very easy’, ‘easy’, ‘difficult’, and ‘very difficult’ [37, 38]. The original questionnaire is validated [38], frequently used, and has been translated into various languages [3]. The Swedish version has been tested for psychometric validity and reliability with satisfying results) [39–41]. Sw-eHEALS is the Swedish version of eHEALS which consists of eight questions about the level of electronic HL (eHL) [42]. Each question has a 5-point Likert-like scale with the answers: ‘strongly disagree’, ‘disagree’, ‘neither’, ‘agree’, and ‘strongly agree’ [42]. Psychometric testing reports satisfying reliability and validity in general [42, 43] and for the Swedish version [36].

Independent variables were lifestyle habits, overweight/obese, health status, pain distribution, and radiographic KOA. Lifestyle habits were assessed via the Swedish National Board of Health and Welfare’s questions about living habits (physical activity, diet, tobacco, and alcohol) [44]; overweight/obese via BMI and waist circumference (obesity classified in accordance with International Diabetes Federation (IDF), as waist circumference ≥ 94 cm in men and ≥ 80 cm in women [45]); health status via the Swedish validated Short-Form General Health Survey (SF-36) questionnaires [46], pain distribution using a pain mannequin [47], and rKOA via x-rays according to the Ahlbäck classification of knee osteoarthritis [48].

The SF-36 questionnaire has eight different subscales: physical functioning; role function—physical aspect; bodily pain; general health perception; vitality; social functioning; role function—emotion aspect; and mental health. Each subscale’s score ranges from 0 to 100, where a higher score indicates better health status [46, 49].

The participants were divided into three groups based on the pain mannequin: no chronic pain, chronic regional pain, and CWP according to the 2019 criteria for CWP [50].

#### Qualitative data

Individual semi-structured interviews were conducted between December 2020 and May 2021. The participants choose the interview setting; either by telephone (*n* = 12), via web-based videoconferencing (*n* = 5), or in-person (*n* = 2) at a Research and Development Centre. The interview guide was based on the HLS-EU questionnaire and health literacy matrix [32] (Additional file 5). All interviews were initiated with open-ended questions such as “How do you find health information?“, “How do you assess the credibility of the health information?“, and “How do you make decisions based on the information you obtain?“. The interviewers followed the participants’ reasoning, and to obtain depth in the data, the participant received follow-up questions such as “Please, can you tell me more about. . .?“ or “How do you mean…?“. The interviews were audio-recorded and transcribed verbatim.

### Data analysis

#### Statistics

The HLS-EU-Q16 SE questionnaire was analysed based on the instrument’s recommendations [38]. The answer options ‘very easy’ and ‘easy’ were dichotomised to ‘easy’ (score: 1), and the answers ‘difficult’ and ‘very difficult’ to ‘difficult’ (score: 0). The total score ranges from 0 to 16 points, and a higher score indicates a higher level of GHL. The following cut-off values were used: score 0–8 indicates an inadequate level of GHL; 9–12 problematic level; 13–16 sufficient level. The three cut-off points were then further dichotomised into a limited level (inadequate + problematic, 0–12 points) and a sufficient level (13–16 points). In the Sw-eHEALS questionnaire, the total score ranges from 8 to 40 points, where a higher score indicates a higher level of eHL. The following cut-off points were used: 8–20 points indicated an inadequate level of eHL; 21–26 problematic; 27–40 sufficient [36]. These three cut-off points were dichotomised into a limited level (inadequate + problematic, 8–26 points) and a sufficient level (27–40 points). GHL and eHL were merged into one variable, ‘HL’, where sufficient HL was defined as having a sufficient level of GHL and eHL.

Data were not normally distributed, and non-parametric statistics were used. For analyses between different groups, Mann-Whitney U-tests were used for interval data and the Chi^2^ test was used for nominal data. Univariate logistic regression was used to analyse associations, and variables with a *p*-value ≤ 0.25 were included in the multivariate analysis [49], controlled for age. Two models were conducted. In model 1, the variables age, education, diet, alcohol intake, pain distribution, and rKOA were included. In model 2, general health was added. All analyses were performed in IBM SPSS 28 (released 2021; IBM Corp., Armonk, NY, USA).

### Qualitative content analysis

The interviews were analysed using manifest qualitative content analysis using an abductive approach, according to Graneheim and Lundman [51–53]. Qualitative content analysis is a systematic approach to making valid inferences from verbal or written data to gain a richer understanding of a phenomenon and is used to develop knowledge of human health and illness experience [54]. The interviews were listened to and read through several times by the first author (CS) to obtain an overview of the material. The analysis began with the extraction of meaning units related to the study’s aim. In total, 409 meaning units were extracted (participants with sufficient HL = 208 units; limited HL = 204 units). Then the de-contextualisation phase began by condensing the meaning units into descriptive codes close to the text [53]. At first, the codes were sorted according to their interrelationships and differences based on the four domains in the HLS-EU health literacy matrix: access, understanding, evaluation, and use of information relevant to health (see Additional file 1) [32], or a range of domains if they included multiple components, creating a first division [51, 52]. This phase had a low level of abstraction and interpretation [53].

Next, the level of interpretation and abstraction increased when the codes under each domain were re-contextualised and sorted into sub-categories, and categories were formed [53]. This process continued back and forth (CS, AKW, IL) until a consensus on the material was reached, together with a discussion in the research team to increase credibility [52, 53]. During this process, the interpretation and level of abstraction increased but remained manifest [53]. The domains of *access* and *apply health information* remained intact when the final categories emerged, but codes in *understand* and *evaluate* came to be combined into one category, resulting in a final sum of three categories and seven sub-categories.

### Ethics

The studies have been approved by the Swedish Ethics Review Authority (Dnr 2016/816; 2017/205; 2020–04489). The study adhered to the Helsinki Declaration [55], and all participants had received information about the study prior to participation, including information about the study’s aim and method. They were also informed about their voluntary participation and the possibility of withdrawing at any time. All participants signed a written informed consent document.

## Results

### Quantitative data

#### Descriptive statistics

Out of the 221 participants included, 71% reported sufficient HL –reporting both sufficient GHL and eHL (separately: 79% sufficient GHL, 81% sufficient eHL). Table 2 presents descriptive information and differences between the groups with sufficient and limited HL. Those with sufficient HL reported higher education and found the internet (for accessing health information and making informed health-related decisions) more usable and important compared to the group with limited HL (*p* < 0.001). No differences were found between the groups regarding lifestyle habits, except that more had a healthy diet in the group with sufficient HL (29% vs. 14%, *p* = 0.013).

**Table 2:** Descriptive statistics for the whole sample and the groups of sufficient and limited health literacy (HL). Presented as numbers and percentages: n (%), unless otherwise stated

	Total, n (%) *n* =221	Health literacy, n (%)		***p***-value
		Sufficient *n* =156	Limited *n* =65	
Age, mean years (sd)	56 (8)	55 (8)	57 (8)	0.580
Gender,				0.533
Women / men	147 (66.5) / 74 (33.5)	106 (68) / 50 (32)	41 (63) / 24 (37)	
Education,				<0.001
Compulsory school	34 (15.5)	15 (10)	19 (29)	
Secondary	87 (39.5)	63 (40)	24 (37)	
University	100 (45)	78 (50)	22 (34)	
Native-born / foreign-born	193 (88) / 27 (12)	136 (88) / 19 (12)	57 (88) / 8 (12)	1.000
In a relation / living alone	173 (78) / 48 (22)	121 (78) / 35 (22)	52 (80) / 13 (20)	0.725
Usability of internet use, Not useful / useful	52 (24) / 168 (77)	20 (13) /136 (87)	32 (50) / 32 (50)	<0.001
Importance of the internet, Not important / important	47 (21) /173 (79)	23 (15) / 133 (85)	24 (37.5) / 40 (62.5)	<0.001
GHL, median (IQR)	16 (13-16)			
Sufficient level / limited level	176 (79) / 46 (21)			
eHL, median (IQR)	32 (28-35)			
Sufficient level / limited level	175 (81) / 41 (19)			
BMI^a^				0.168
Normal	100 (46)	75 (48)	25 (40)	
Overweight	71 (32)	52 (33)	19 (30)	
Obese	48 (22)	29 (19)	19 (30)	
Waist circumferenc^b^				
Obese / not obese	176 (82) / 38 (18)	124 (82) / 27 (18)	52 (82.5) / 11 (17.5)	1.000
Meets recommendations for physical activity^c^	126 (57)	87 (56)	39 (60)	0.655
Healthy diet^d^,	54 (24)	45 (29)	9 (14)	0.013
Smoker,	16 (7)	11 (7)	5 (8)	0.526
Snuff user,	10 (4.5)	8 (5)	2 (3)	0.403
Alcohol intake,				0.105
<1 unit/week	83 (38)	63 (41)	20 (31)	
1–4 units/week	99 (45)	70 (45.5)	29 (44.5)	
≥5 units/week	37 (17)	21 (13.5)	16 (24.5)	
Pain group,				0.166
NCP	41 (19)	28 (18)	13 (2)	
CRP	146 (66)	68.8 (70)	35 (58.5)	
CWP	33 (15)	19 (12)	14 (21.5)	
rKOA^e^	75 (35)	47 (31)	28 (44)	0.085

Sufficient HL was defined as having a sufficient level of general HL and electronic HL

*Sd *Standard deviation, *BMI *Body mass index, *GHL *General health literacy, *IQR *Interquartile range, *eHL *Electronic health literacy, *NCP *No chronic pain, *CRP *Chronic regional pain, *CWP *Chronic widespread pain, *rKOA *Radiographic knee osteoarthritis

^a^Normal BMI =18.5–24.9 kg/m^2^; overweight =25.0–29.9; obesity ≥30.0. ^b^Obese =classified in accordance with IDF as waist circumference ≥94cm in men and ≥80cm in women. ^c^WHO recommendations: 150–300 minutes of moderate intensity and/or 75–150 minutes of vigorous intensity. ^d^Vegetables and fruit every day, fish 2/week, breakfast most days, pastries a few times/week. ^e^Having a score ≥1 on the Ahlbäck scale for knee osteoarthritis

The group with sufficient HL reported better health in the SF-36 sub-scales: role function—physical aspect (mean difference *δ* = 5.2; *p* = 0.035) and general health perception (*δ* = 7.5; *p* = 0.011) than the group with limited HL (see Additional file 2).

#### Associations with health literacy

In the univariate analysis, higher education, a healthy diet, < 1 unit of alcohol/week, and better general health were associated with sufficient HL (Table 3). Contrary, worse general health and higher alcohol consumption were associated with limited HL (see Additional file 3).

**Table 3:** Univariate logistic regression analysis of associations with sufficient health literacy (HL). Presented as odds ratio (OR) and 95% confidence interval (CI)

	Sufficient health literacy		
	n	OR (95% CI)	***p***-value
Age	221	0.98 (0.94–1.02)	0.265
Gender	221		
Man		1	
Women		1.24 (0.68–2.27)	0.485
Education	221		
Compulsory school		1	
Secondary		3.33 (1.46–7.58)	0.004
University		4.49 (1.97–10.26)	<0.001
Waist circumferenc^a^	214		
Obese		1	
Non-obese		1.03 (0.48–2.23)	0.942
Physical activity^b^	221		
Does not meet recommendation		1	
Meets recommendation		0.84 (0.47–1.51)	0.563
Diet^c^	221		
Less healthy diet		1	
Healthy diet		2.52 (1.15–5.53)	0.021
Smoker	219		
Yes		1	
No		1.11 (0.37–3.33)	0.853
Snuff user	220		
Yes		1	
No		0.60 (0.12–2.89)	0.521
Alcohol intake	219		
≥5 units/week		1	
1–4 units/week		1.84 (0.84–4.02)	0.126
<1 unit/week		2.40 (1.06–5.64)	0.037
General health *(Scoring 0–100, worst-best)*	216	1.02 (1.01–1.04)	0.011
Pain distribution	220		
CWP		1	
CRP		2.09 (0.96–4.58)	0.064
NCP		1.59 (0.61–4.12)	0.342
rKOA^d^	216		
Yes		1	
No		1.74 (0.95–3.17)	0.072

Sufficient HL was defined as having a sufficient level of general HL and electronic HL

*CWP *Chronic widespread pain, *CRP *Chronic regional pain, *NCP *No chronic pain, *rKOA *radiographic knee osteoarthritis

^a^Obese =classified in accordance with IDF as waist circumference ≥94cm in men and ≥80cm in women. ^b^WHO recommendations: 150–300 minutes of moderate intensity and/or 75–150 minutes of vigorous intensity. ^c^Vegetables and fruit every day, fish 2/week, breakfast most days, pastries a few times/week. ^d^Having a score ≥1 on the Ahlbäck scale for knee osteoarthritis

In the multivariate model controlled for age: higher education, a healthy diet, and < 1 unit of alcohol/week, and chronic regional pain were associated with sufficient H.L. Only higher education and better general health remained associated when adding general health to the analysis (Table 4).

Better general health (OR 0.97, CI 0.95–0.99) and chronic regional pain (OR 0.39, CI 0.15–0.97) were negatively associated with limited HL, whereas a higher alcohol consumption (1–4 units/week OR 2.50, CI 1.05–5.97; ≥5 units/week OR 3.26; CI 1.20–8.89) were positively associated with limited HL (see Additional file 4).

**Table 4:** Two models with multivariate logistic regression analysis of associations with sufficient health literacy (HL). Model 1 included age, education, diet, alcohol intake, pain distribution, and rKOA. In model 2, general health was added. Presented as odds ratio (OR) and 95% confidence interval (CI)

	Sufficient health literacy				
		Model 1		Model 2	
	n	OR (95% CI)	***p***-value	OR (95% CI)	***p***-value
Age	221	0.99 (0.95–1.04)	0.778	0.99 (0.95–1.04)	0.749
Education	221				
Compulsory school		1		1	
Secondary		4.39 (1.75–10.99)	0.002	4.85 (1.90–12.40)	<0.001
University		4.65 (1.83–11.84)	0.001	5.41 (2.06–14.22)	<0.001
Diet^a^	221				
Less healthy diet		1		1	
Healthy diet		2.53 (1.07–5.98)	0.035	2.14 (0.89–5.15)	0.091
Alcohol intake	219				
≥5 units/week		1		1	
1–4 units/week		1.94 (0.82–4.59)	0.131	1.66 (0.68–4.05)	0.263
<1 unit/week		2.51 (1.00–6.29)	0.049	2.48 (0.97–6.34)	0.058
Pain distribution	220				
CWP		1		1	
CRP		2.81 (1.16–6.80)	0.022	1.85 (0.70–4.87)	0.213
NCP		1.81 (0.63–5.15)	0.269	0.91 (0.27–3.02)	0.875
rKOA^b^	216				
Yes		1		1	
No		1.37 (0.69–2.74)	0.371	1.35 (0.66–2.76)	0.417
General health *(Scoring 0–100, worst-best)*	216			1.02 (1.00–1.04)	0.019

‘Sufficient HL’ was defined as having a sufficient level of general HL and electronic HL. ^a^ Vegetables and fruit every day, fish 2/week, breakfast most days, pastries a few times/week. ^b^Having a score ≥1 on the Ahlbäck scale for knee osteoarthritis

*CWP* Chronic widespread pain, *CRP *Chronic regional pain, *NCP* No chronic pain, *rKOA *Radiographic knee osteoarthritis

#### Qualitative results

**Fig. 1:**
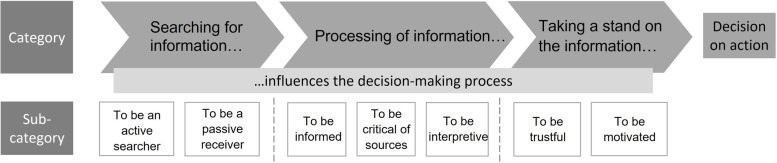
Overview of the results exploring the experiences of health literacy in individuals with knee pain, presented with three categories and seven sub-categories illustrating the process towards a deciding on action

The qualitative content analysis resulted in three categories and seven sub-categories exploring individuals with knee pains experiences of health literacy and phases in the decision-making process concerning healthcare, disease prevention, and health promotion. The emerging categories were *searching for information influences the decision-making process*, with the sub-categories: to be an active searcher and to be a passive receiver; *processing of information influences the decision-making process*, with the sub-categories to be informed, to be critical of sources, and to be interpretive; and *taking a stand on the information influences the decision-making process*, with the sub-categories to be trustful and to be motivated. See Fig. 1.

### Searching for information influences the decision-making process

The category ‘Searching for information influences the decision-making process’ is further explained by two diverse interactions: being an active searcher and a passive receiver.

### To be an active searcher

Health information was actively searched for when in need or due to interest. The sources varied, and the internet/Google, TV shows, peers, health insurance information, health care professionals, and non-qualified health personnel were expressed as sources. Participants spoke of finding inspiration on social media or training apps and searched for information on different sources to find something that would help their current health situation.

Participants with sufficient HL valued information from younger health personnel, stating they probably had more updated knowledge. Additionally, they searched for information on authorities’ websites and claimed it as positive to be recurrently reminded of already-known health information.

*It is often the same information that appears, and some information you already know, such as getting outside, getting fresh air, and being physically active, is good for you. It’s really not much new information. I suppose I search for information because I don’t feel so well and want to feel better.* (Participant no. 16, woman, sufficient HL).

Participants with limited HL expressed that negative information had the most impact, and they searched for information that could increase their health status. They further expressed that finding information was an individual responsibility, but it was difficult to find the right information. They expressed that double-checking information with health care personnel facilitated their search and that information about health risk factors helped educate them.

*If I get information about something that has been negative, then I acknowledge it better. I need to be better than that. So, I see it differently in some way “oh well, how have they behaved? I shouldn’t do that”. I learn more from a mistake than from success, I think.* (Participant no. 9, male, limited HL)

### To be a passive receiver

Health information was expressed as infinitely available, where information via, e.g., conventional media (newspapers, TV news, radio etc.) was passively received. If the flow of information was too great, it resulted in even more passivity and, at times, ignorance. Information was received passively at workplaces, either in a formal context (having an occupation in health or by receiving lectures and safety briefings) or informally, through discussions with co-workers. Some avoided searching for health information due to having no interest or need. Others expressed receiving information from their wife.

*Information flies by. I have seen that there are osteoarthritis schools and everything you can see online. Most often, I see notices in the newspapers, “this is how you deal with your arthritic knees”. I would say that I have not delved into it. I thought at some point that maybe I should try an osteoarthritis school. I thought about whether I should use the money I have from work for something like that, but I’ve mostly chosen to listen to my body instead.* (Participant no. 18, woman, limited HL)

There were some differences between the participants with limited and sufficient HL. The group with sufficient HL argued that they already knew enough about health and did not need additional information or not need information due to good health status, hence, mainly receiving health information passively. In contrast, the participants with limited HL avoided reading or listening to negative information about a personal situation (e.g., odds of successful surgery).

*If I get it [information], it’s advertising on TV, advertising in e-mail on the phone about everything. If I want to find something about health, it’s not difficult. Then maybe I don’t believe in everything, but there is an incredible amount of information. So, I’m almost fed with it. I feel like I can’t buy everything.* (Participant no. 4, woman, sufficient HL)

### Processing of information influences the decision-making process

Another interaction with health information is the processing of information which influences the decision-making process. The ability to understand affected the evaluation of information and, thus, the decision-making process, which is further explained by being informed, critical of sources, and interpretive. Additionally, processing information involved a degree of personal evaluation.

#### To be informed

Participants expressed not giving much thought to information. They received the information, and either trusted it or rejected it at once, no matter the source (e.g., social media or healthcare personnel). The evaluation was based on personal feelings and what suited them. No systematic evaluation was described. Participants further described that this way of processing information was based on life experience and that a ‘gut feeling’ was key. But receiving various and conflicting information from healthcare personnel made it difficult to understand and evaluate the information, thus stopping the decision-making process. Contrary, receiving adequate and clear information facilitated the decision-making process.

*Interviewer: you listen to what suits you?*

*P14: Yes, I think so, actually. There can be things like “this is how you should do it”, but if I don’t think it looks like fun, I don’t care. I probably have my own way based on things I’ve learned over the years, and I stick to it. Well, now that you say it, maybe I’m not that open-minded to taking in that many new things at all really.* (Participant no. 14, male, sufficient HL)

Participants with sufficient and limited HL used this simple information process, but the group with limited HL was more evident. They expressed difficulty in understanding health information and what counted as facts and not just opinions. The non-qualified health industry was untrustworthy, and there were feelings of being misled. Some expressed that it was problematic with new communication channels in the healthcare, such as chatting or texting, as well as navigating on authorities’ webpages. It was also difficult to understand and evaluate information from health care personnel if one had previously been questioned or mistrusted when searching for help.

*Sometimes I blindly believe what I read. Then I start googling it even more. Then it’s that person who said that or this person who said this, so I feel like, can I really trust anything online? No. So then I go to healthcare web pages or similar pages, where something completely different is written. Then I think, what is actually true?* (Participant no. 19, woman, limited HL)

### To be critical of sources

The participants described a more thorough process by being critical of the source. When receiving information, the process went through a source of criticism and then personal evaluation. Uncertain sources defined by participants with sufficient and limited HL, included the internet or Google, social media, newspapers, and commercials. Participants expressed being wary of reading health information on these sources, and that the information could serve as inspiration rather than fact. Still, the personal evaluation was present, and in the end, the participants mainly recognised the information that felt relevant to them.

*Most of what I read online, I read with some caution. It depends a little on what kind of articles are in newspapers and such. It’s difficult to know how much to believe in different things. I was about to say that I believe in what I want to believe in.* (Participant no. 6, woman, limited HL)

Neither participants with sufficient nor limited HL stood out when being critical of sources. Information from experts was preferred over that coming from journalists, relevant and clear information was desired, and common sense was used when evaluating information. Health information was considered important, and with time, participants learned which sources were trustworthy and which required more criticism.

*Yeah, I mean, she [a friend] is doing a lot of things, and I’m like, “no, this sounds too good to be true”, and then I don’t fall for it either. It must be things that I feel I could do. It must sound credible, and it must not be too vague. I won’t eat a bunch of substances and stuff just because someone says this is good for you. Because you never know.* (Participant no. 15, woman, sufficient HL)

### To be interpretive

Participants described a further comprehensive process of information by being sceptical, listening to several different sources, going back and forth, and trying to generate an overall picture by being interpretive. Having a comprehensive and iterative evaluation process was most common in participants with sufficient HL. A systematic evaluation was applied, going back and forth between information, understanding information they received, and criticising sources. If the information was lacking, participants went back to an active search for more information to derive meaning and generate an overall picture before continuing the process to decide on action.

*Yes, there is a lot on the internet, but it’s not like everything can be trusted. Interviewer: Why not? P12: No, you have to have some source criticism. It’s like checking the weather. I have 4 or 5 apps to compare between, and then I get an average. I do the same if I Google. You can’t just read one article. So, if you Google something, you have to look at several different ones, so you can see the whole of it.* (Participant no. 12, male, limited HL)

Evidence and research-based information formed the foundation of the decision-making process, as well did an understanding of the complexity and multi-faceted nature of health. Information that seemed credible on the surface, or because an informant had a seemingly trustworthy title (e.g., physiotherapist, researcher, etc.) was still evaluated with some level of criticism. Part of the high information process was after deciding on an act, participants went back and further evaluated their choice, modifying or finding more information as needed.

*I need to see who it is that has released the information. If it was a post on Facebook, it is perhaps not so credible. Is there any research behind it? So, it’s not just speculation. Then, as I said, I have a pretty good idea myself. I notice pretty quickly if it’s good stuff or not. Interviewer: You have your education behind you?*

*P10: Yes.* (Participant no. 10, male, sufficient HL)

### Taking a stand on the information influences the decision-making process

Taking a stand on the information influenced the decision-making process and the individual’s standpoint was based on trust in the information or the informant and the participant’s motivation.

### To be trustful

Personal contact was expressed as important for feeling trust about information. Trust in the informant could increase the possibility of action (e.g., seeking medical advice or trying a new exercise to reduce pain), but it could also decrease the level of criticism of the source.

Participants with sufficient HL expressed the importance of the informant being trustworthy and was based on expertise, occupation, or personal lived experience (e.g., having had knee surgery, etc.). Among participants with limited HL, personal contact (e.g., physical meetings with healthcare personnel or information from peers) was expressed as important regarding trustworthiness. Feelings of trust were also more or less independent of the information’s reliability.

Participants expressed frustration that healthcare professionals did not provide adequate information, resulting in reduced trust and difficulties in accepting, e.g., information from a general practitioner regarding health outcomes and treatment.

*Well, it’s quite clear if a friend of mine who I hold dear says something, I listen to it. I have a lot of good friends who are physicians and chiropractors and so on, so if they would say something, people that I have high confidence in saying something, then I would trust it blindly. Because then I know that it is substantiated and that they have a good grasp of this, and these people wouldn’t say anything if they didn’t believe it themselves. So, it means a lot to me. I listen to that. (Participant no. 14, male, sufficient HL)*

Relatedness to the information increased trust and the possibility of action. With no personal recognition, the information was deemed irrelevant and ‘not for me’, regardless of whether the informant was a peer, a stranger on the internet, or a health professional. Previous experience with health care affected the feelings of trust in the information and informant. Having received good care, the sense of trust was high. But having received poor care, this trust was lost, which resulted in difficulty applying and acting on the information. Trust in oneself to make the right decision also had an impact on the decision to act.

*In general, I am a little sceptical about medicines. I want to try, above all, to start with a little healthier stuff. I’d rather not go on medication. I haven’t even dared to take cortisone shots for my knees because I think, well, it might not be good for me. But I have heard of many who can manage on it for several months.* (Participant no. 1, woman, limited HL)

### To be motivated

Finding, processing, and understanding information, with the conclusion that, e.g., a behaviour change was needed to increase health/improve their current state, was not enough to decide on the proposed action. The participants expressed reluctance to change, and either accepted their current health status or sought other information and possible health-promoting actions. Lack of motivation led to decisions based on old habits, often not wanting to act or to change the current way of living. Additionally, the participants with limited HL expressed a lack of motivation resulting in ignoring healthcare follow-ups, since their experienced health status was deemed ‘okay’.

*I guess I’m a bit lazy in some way too. It hasn’t been that I’ve gained interest in it. I haven’t felt like it was my thing either, but later on, it is possible that I would. But no, touch wood, I’ve been good for a while now as long as I’m in motion somehow. Sometimes things resolve themselves.* (Participant no. 22, male, limited HL)

With enough motivation, the participant could decide to act on the information and make an attempt, no matter what was suggested. Seeing or feeling some effect was necessary to stay motivated and pursue an activity to promote health or prevent disease. Despite previous information, the motivation was lost if there was no result in a self-defined time frame. Some participants expressed a non-interest in change due to already-good health status.

*But I wonder if it isn’t the case that what I think is fun is easier to feel comfortable with. Then I may find it easier to try something new and change myself. What I think is difficult and challenging, in those cases, I want to continue with my old, regular ways. If I already think there is a bit of a hard feeling about it, then it will be more challenging if I have to change something too. As I said, it is not simple. (participant no. 6, woman, limited HL)*

### Mixed results

The main findings were that two-thirds of the individuals with knee pain reported sufficient HL. Better general health was associated with sufficient HL. Experiences of good health contributed to individuals describing themselves as passive recipients of health information without a need for additional health information.

Individuals with sufficient HL had higher education and reported, in the questionnaires, that the internet was a useful source and important when searching for health information. Contrary, individuals with limited HL reported it was not useful and challenging to navigate the wealth of information. In the qualitative interviews, individuals in both groups described how they actively searched for health information from the internet and other sources. Information processing varied, and both groups expressed the comfort of being informed but still critically reviewing the sources before interpreting and taking a stand on the information.

**Fig. 2:**
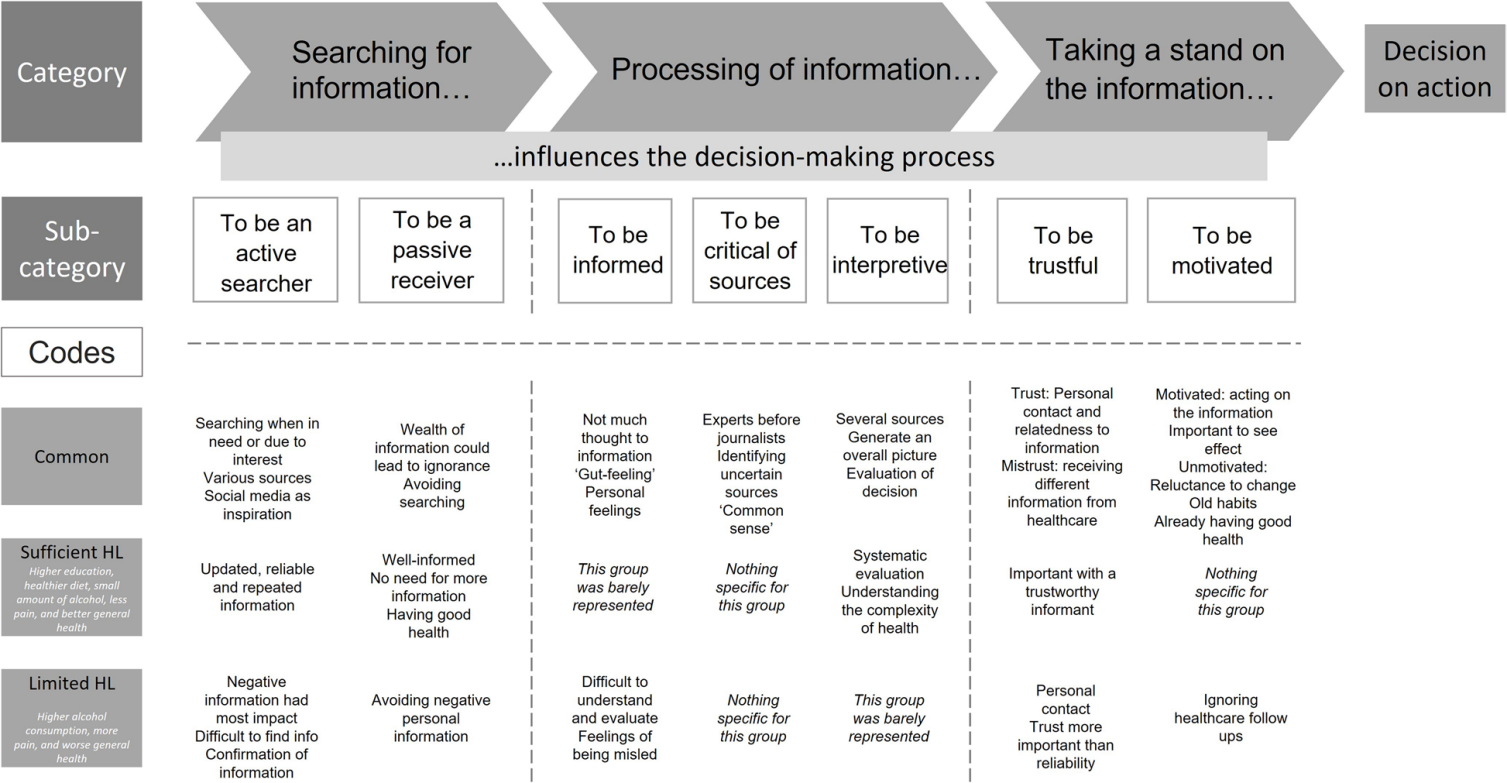
Overview of the mixed results exploring associations and the experiences illuminating health literacy in individuals with sufficient and limited health literacy, presented with the three categories, seven sub-categories and codes illustrating the process towards a decision on action

Individuals with sufficient HL reported having a healthy diet, but no other differences regarding lifestyle habits were found between the groups. The qualitative findings revealed different phases in the decision-making process, including searching, processing, and taking a stand on health information. Many similarities between the groups were found, and individuals with sufficient and limited HL were represented in most sub-categories (see Fig. 2). The groups differed most in the processing of information where individuals with sufficient HL were overrepresented in the sub-category ‘to be interpretive’, having a comprehensive and systematic approach when understanding and evaluating information. Contrary, individuals with limited HL were overrepresented in the sub-category ‘to be informed’, not giving too much thought to information and leaning on personal feelings such as the ‘gut feeling’.

## Discussion

This study explored the level of HL and its associations with health status, lifestyle habits, chronic pain, rKOA, and experiences illuminating HL among individuals with knee pain. The study’s mixed methods design provides important empirical insights on HL in individuals with knee pain from both quantitative and qualitative datasets.

### Level of health literacy

The findings show that although a majority of participants reported sufficient HL (71%), a significant proportion reported limited HL (29%), highlighting a possibly disadvantaged group among individuals with knee pain. There is heterogeneity in previous research regarding HL measurements and pain criteria, with a range of 17–82% reporting limited HL amongst individuals with pain [30]. The prevalence of limited HL in the general population in Europe differs from 29 to 62% [4]. But a recent study from Sweden found results similar to the current study, where approximately a third reported limited HL [56].

Moreover, participants with sufficient HL had a higher level of education than the group with limited HL. The association between the level of HL and education is well-known from previous studies [4].

### Health literacy and lifestyle habits

In this study, more individuals with sufficient HL reported having a healthy diet. No other differences regarding lifestyle habits were found between the groups. A healthy diet and low intake of alcohol were the lifestyle habits associated with sufficient HL. In a newly published systematic review, HL and self-management in individuals with pain were studied, and the authors found no published studies including lifestyle factors other than physical activity [30]. Based on a graphical model, the results stated no association between HL and exercise [57]. It is evident that more research is needed on the association between HL and modifiable lifestyle factors included in the self-management recommendations for individuals with rKOA and/or chronic pain.

The participants in this study expressed an unwillingness to adjust or make a behavioural change, despite communicating that they understood the benefits of the change. Individuals with limited HL could also ignore healthcare follow-ups. Lack of motivation, regardless of perceived knowledge, led to no action regarding healthy lifestyle habits. On the contrary, having enough motivation could lead to action, but with no visible results, motivation was lost, as was the healthy habit.

Self-management is important to prevent worsening pain problems and maintain or improve health status [18]. Additionally, HL is essential for facilitating self-management [8]. But the current study’s results state that the general pursuit of healthy lifestyle habits is rather low in both groups, and that having sufficient HL is not enough for a behavioural change.

The HLS-EU health literacy matrix explains the domain ‘*apply information’* as “the ability to make informed decisions on medical issues/on risk factors for health/on health determinants in their social and physical environment” [32]. A logical question to ask is whether an individual indeed has understood the information, including pros and cons, consequences, etc., in order to have the ability to make such an informed decision. On the other hand, a possible explanation could be the difficulty in comprehending the information that continuous unhealthy lifestyle habits are indeed risk factors for rKOA and chronic pain development. In addition, there can be a disconnect between the individual’s experiences, beliefs, and attitudes about the risk factors involved [8], especially a lack of physical activity and/or being overweight [17, 18].

Sufficient HL might impact the pursuit of healthy lifestyle habits among individuals with knee pain, but it is not a single factor. The somewhat contradictory results indicate that more research is needed to understand the complexity of HL and self-management to promote healthy lifestyle habits in individuals with onset symptoms of rKOA and chronic pain.

### Health literacy and health status, chronic pain, and rKOA

In the present study, there were no significant differences regarding obesity, pain distribution or prevalence of rKOA between groups with sufficient and limited HL. Participants searched for information to improve their health status or prevent disease. Other participants claimed no interest in health information due to good perceived general health. Furthermore, reporting good general health, despite having unhealthy lifestyle factors or chronic pain, was also associated with sufficient HL. These results suggest that the HL level is related mainly to perceived health.

Previous studies suggest no difference in the level of HL among individuals with chronic pain or without chronic pain [30]. A proposed explanation was that individuals with chronic pain are more exposed to educational opportunities and, therefore, report a higher level of HL. Individuals with chronic pain have indeed reported higher health care utilisation—i.e., *possible educational opportunities*—than individuals with no chronic disease [58]. A recent study among individuals with chronic pain found that the level of eHL was negatively associated with depression and anxiety, and the level of self-efficacy was the explanatory factor [25]. In addition to low self-efficacy, individuals with ill health may receive inadequate information from healthcare and therefore report struggles and limited HL. For example, participants in this study expressed a difference in trust towards health information based on previous interactions with healthcare personnel. The feeling of trust was high if participants had had positive experiences in health care, but low if they had had negative experiences. In turn, the amount of trust affected the possibility of acting on the information.

Previous research found similar results among older adults, where participants did not believe in health information before trust was built [59]. Additionally, trust seemed to impact the willingness to perform physiotherapy. Misinterpretation and lack of knowledge about KOA are negatively associated with the level of physical activity and other health-promoting behaviours [60], and there can be evident discord between patients’ and physicians’ experiences of KOA. For example, patients expressed wanting their condition to be considered more severe and important than what physicians expressed [61]. Individuals with rKOA have also described that they wished they had received information about the development of rKOA and its preventive measures at an earlier stage of disease progression [62].

It is possible that individuals with ill health have higher HL demands compared to individuals with less contact with health care. Much health information is focused on medical knowledge translation without any individual context, which can make it difficult for individuals to translate into their own context [63]. Another possibility is that the quantitative results represent how well the individual’s skills match the demands or expectations of, for example, the healthcare worker providing the information [64]. Individuals with perceived good general health may find the HL demands effortless, and therefore feel no need to receive more information and, as a result, report sufficient HL. The results of sufficient or limited HL might thus not be fully representative when comparing individuals with perceived good general health with individuals with perceived ill health.

### Experiences of health literacy

The qualitative results revealed a deeper understanding of how individuals with knee pain experience HL. Health information was either actively searched for or received passively. Ways of processing this information ranged from being informed to being interpretive, and the standpoint of the information affected the final decision of whether to act. In this study, it was not possible to fully separate the results into the domains *understand* and *evaluate* from the HLS-EU-health literacy matrix [32]. Instead, these were merged into one category: *processing of information influences the decision-making process.* According to Lonergan’s theory of knowledge, understanding occurs through questioning based on previous information, experiences, and personal opinions [65]. Evaluating is instead the process of weighing the information against previous knowledge, etc. Separating understanding and evaluating can therefore be difficult in an interview context.

There is a wealth of health information; something which participants with both sufficient and limited HL expressed. Conflicting information made it harder the participants to continue the HL process towards a decision to act. WHO has discussed this issue, and stated that the large volume of information makes it harder to easily or properly interpret and appraise information [8]. Furthermore, participants with sufficient HL expressed the internet as useful if they remained cautious and critical of the source. On the contrary, participants with limited HL expressed difficulties navigating health authorities’ web pages, using digital tools such as chatting with health personnel, and finding relevant information. These results were also visible in the quantitative results, where the participants with sufficient HL found the internet useful and important for accessing health information and making decisions, in contrast to the participants with limited HL.

The study’s results are in line with previous research. For example, a recent study found that individuals with sufficient HL were more prone to search for information on an online national health information portal (by the Swedish national health care system) compared to individuals with limited HL [56]. Another study found that individuals with chronic conditions reported slightly higher eHEALS scores and were more prone to read, ask, and act upon information found on the internet than those with no chronic condition [66]. The study also found that participants with chronic diseases were more likely to track health indicators. Another study evaluating an internet-based cognitive behaviour therapy for chronic pain found several difficulties and concluded the importance of test usability in relation to electronic health literacy to achieve the desired effect [67].

Individuals with knee pain and sufficient or limited HL reported many similarities regarding their experiences illuminating HL. These results add to the knowledge of the importance of individualised information so that individuals with knee pain can easily translate it into their context to facilitate health promotion.

### Methodological considerations

A strength of the convergent mixed methods design is that synthesising the quantitative and qualitative results generates a more complete understanding of the phenomenon [68], in this case, HL in individuals with knee pain. Nevertheless, using a convergent mixed methods design comes with challenges and requires a well-planned study design to ensure that the different datasets target the same concepts [68]. In this study, both the questionnaire for general HL and the interview questions were based on the same HL definition [3] and theoretical framework (HLS-EU-health literacy matrix [32]), which is therefore considered a methodological strength. The co-authors have great competence regarding subject and methods and to gain trustworthiness, all researchers contributed to different parts of the study according to their expertise [68].

The non-responders were younger and had higher levels of education, which can be a limitation and negatively affect the generalisability of the results. But since no other differences were found, and age and HL were not associated in this group, it is still considered possible to generalise the results to adult individuals with knee pain. However, the cross-sectional design cannot draw any causal conclusions, and future longitudinal studies are needed.

In qualitative studies, credibility, dependability, conformability, and transferability are used to evaluate trustworthiness [52, 69]. The credibility of the present study was strengthened by the method used to collect and analyse data suited to answering the research question. The study’s credibility was further strengthened by the selection of participants, who represented various characteristics generating various experiences and aspects [51, 52]. However, some participants had difficulties talking and reflecting on the different aspects of HL (access, understand, evaluate, and use health information), which can be a limitation and possibly decrease the study’s credibility. Still, clarifications and follow-up questions were asked to increase credibility.

The two interviewers were new to the interview process but were supervised by an experienced researcher (IL) to increase dependability. Additionally, the interviews began with the same question and regular follow-up questions to facilitate the participant’s conversation about their experiences [52]. The main author (CS) continually discussed the analysis back iteratively with AKW and IL before reaching a consensus, strengthening the study’s dependability and confirmability [51, 69]. Confirmability was further strengthened by the various quotations illuminating the participant’s experiences as part of the large number of meaning units [52, 69]. The purposeful selection of a variety of participants strengthens the transferability, and the study’s results can likely be transferred to other adults with knee pain [52].

## Conclusion

The insights gained from this study’s results state that HL is a piece of the puzzle in health promotion and self-management. There is a possibly disadvantaged group reporting limited HL among individuals with knee pain, and sufficient HL might have a positive impact on healthy lifestyle behaviours. Having good general health was associated with sufficient HL, but there can be several explanations as to why. This could include, for example, a lack of interest in health information and additional health behaviour.

Both groups reported unhealthy lifestyles and expressed the influence of lack of motivation, suggesting that having sufficient HL is not enough for having or deciding to change to more healthy lifestyle habits. The clinical implications of this study are to be aware that the phases in the decision-making process include searching, processing, and taking a stand on the information. A person-centred approach is essential when providing health information to determine whether the individual is an active or passive recipient, content to be informed or critical of sources or interpretive before the individual is trustful and motivated to decide how to act on the information.

More research on HL is needed to gain knowledge of how best to further develop health promotion in individuals with knee pain.

### Supplementary Information

**Additional file 1.**

**Additional file 2.**

**Additional file 3.**

**Additional file 4.**

**Additional file 5.**

## Data Availability

The quantitative datasets used and analysed during the current study are available from the corresponding author at a reasonable request. The qualitative dataset generated and analysed are not publicly available due to ethics approval for the study that requires that the transcribed interviews are kept in locked files, accessible only to the researchers.
