# Supplementary material for: Health literacy in individuals with knee pain—a mixed methods study
Source: BMC Public Health. 2023 Aug 29;23:1656. doi: 10.1186/s12889-023-16585-9 (PMC10463821; doi:10.1186/s12889-023-16585-9)
Supplement: Supplementary file 1 — Additional file 1. [file 12889_2023_16585_MOESM1_ESM.docx]

**Additional file 1.** The HLS-EU health literacy matrix.

|  | **Access/find/obtain information relevant to health** | **Understand information relevant to health** | **Appraise/judge/evaluate information relevant to health** | **Apply/use information relevant to health** |
| --- | --- | --- | --- | --- |
| **Healthcare** | Ability to access information on medical and clinical issues | Ability to understand medical information and derive meaning | Ability to interpret and evaluate medical information | Ability to make informed decisions on medical issues |
| **Disease prevention** | Ability to access information on risk factors for health | Ability to understand information on risk factors and derive meaning | Ability to interpret and evaluate information on risk factors for health | Ability to make informed decisions on risk factors for health |
| **Health promotion** | Ability to update oneself on determinants of health in the social and physical environment | Ability to understand information on determinants of health in the social and physical environment and derive meaning | Ability to interpret and evaluate information on health determinants in the social and physical environment | Ability to make informed decisions on health determinants in the social and physical environment |
