# Supplementary material for: Health literacy in individuals with knee pain—a mixed methods study
Source: BMC Public Health. 2023 Aug 29;23:1656. doi: 10.1186/s12889-023-16585-9 (PMC10463821; doi:10.1186/s12889-023-16585-9)
Supplement: Supplementary file 2 — Additional file 2. [file 12889_2023_16585_MOESM2_ESM.docx]

**
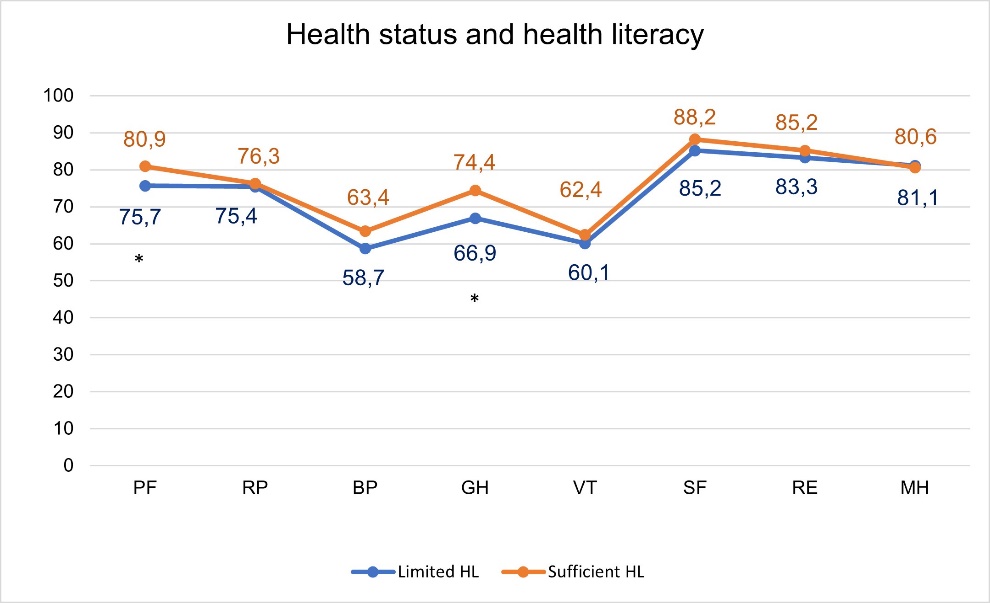
Additional file 2.** Results from the SF-36 subscales in the groups with limited and sufficient health literacy. The sub-scales are physical function (PF); role function – physical aspect (RP); bodily pain (BP); general health (GH); vitality (VT); social functioning (SF); role function – emotional aspect (RE), and mental health (MH). **p* <0.05
