# Supplementary material for: Health literacy in individuals with knee pain—a mixed methods study
Source: BMC Public Health. 2023 Aug 29;23:1656. doi: 10.1186/s12889-023-16585-9 (PMC10463821; doi:10.1186/s12889-023-16585-9)
Supplement: Supplementary file 3 — Additional file 3. [file 12889_2023_16585_MOESM3_ESM.docx]

**Additional file 3.** Univariate logistic regression analysis of associations with limited health literacy (HL). Presented as odds ratio (OR) and 95% confidence interval (CI).

|  | **Limited health literacy** | | |
| --- | --- | --- | --- |
|  | **n** | **OR (95% CI)** | ***p*-value** |
| Age | 221 | 0.99 (0.95-1.04) | 0.740 |
| Gender  Men  Women | 221 | 1  1.15 (0.57-2.32) | 0.706 |
| Education  University  Secondary  Compulsory school | 221 | 1  1.03 (0.49-2.16)  2.18 (0.90-5.26) | 0.945  0.083 |
| General health status (0–100) | 216 | 0.98 (0.97-1.00) | 0.022 |
| Pain distribution  NCP  CRP  CWP | 220 | 1  0.87 (0.30-2.51)  0.59 (0.26-1.33) | 0.800  0.203 |
| KOA^a^  No  Yes | 216 | 1  1.40 (0.71–2.76) | 0.335 |
| Waist circumferenc^e^  Non-obese  Obese | 214 | 1  1.42 (0.55-3.65) | 0.467 |
| Physical activity^c^  Meets recommendation  Does not meet recommendation | 221 | 1  0.96 (0.50-1.87) | 0.908 |
| Diet^d^  Healthy diet  Less healthy diet | 221 | 1  2.44 (0.97-6.12) | 0.058 |
| Smoker  No  Yes | 219 | 1  0.89 (0.24-3.25) | 0.853 |
| Snuff user  No  Yes | 220 | 1  0.97 (0.20-4.74) | 0.971 |
| Alcohol intake  <1 unit/week  1-4 units/week  ≥5 units/week | 219 | 1  1.87 (0.85-4.13)  3.14 (1.23-8.01) | 0.121  0.017 |

*‘Limited HL’ was defined as reporting a limited level of general and/or electronic HL.*

*^a^ Having a score ≥1 on the Ahlbäck scale for knee osteoarthritis. ^b^ Obese = classified in accordance with IDF as waist circumference ≥94cm in men and ≥80cm in women. ^c^ WHO recommendations: 150-300 minutes of moderate intensity and/or 75-150 minutes of vigorous intensity. ^d^ Vegetables and fruit every day, fish 2/week, breakfast most days, pastries a few times/week.*

*CWP, chronic widespread pain; CRP, chronic regional pain; NCP, no chronic pain; KOA, knee osteoarthritis*
