# Supplementary material for: Health literacy in individuals with knee pain—a mixed methods study
Source: BMC Public Health. 2023 Aug 29;23:1656. doi: 10.1186/s12889-023-16585-9 (PMC10463821; doi:10.1186/s12889-023-16585-9)
Supplement: Supplementary file 4 — Additional file 4. [file 12889_2023_16585_MOESM4_ESM.docx]

**Additional file 4**. Two models with multivariate logistic regression analysis of associations with limited health literacy (HL). Presented as odds ratio (OR) and 95% confidence interval (CI).

|  | **Limited health literacy** | | | | | |
| --- | --- | --- | --- | --- | --- | --- |
|  | **Model 1** | | |  | **Model 2** | |
|  | **n** | **OR (95% CI)** | ***p*-value** |  | **OR (95% CI)** | ***p*-value** |
| Age | 221 | 0.99 (0.94-1.03) | 0.526 |  | 0.99 (0.95-1.04) | 0.822 |
| Education  University  Secondary  Compulsory school | 221 | 1  0.78 (0.35-1.75)  2.12 (0.81-5.53) | 0.554  0.126 |  | 1  0.76 (0.33-1.77)  2.27 (0.83-6.16) | 0.526  0.109 |
| Pain distribution  NCP  CRP  CWP | 220 | 1  0.54 (0.23-1.27)  0.97 (0.32-2.96) | 0.158  0.958 |  | 1  0.39 (0.15-0.97)  0.48 (0.13-1.77) | 0.043  0.272 |
| Diet^a^  Healthy diet  Less healthy diet | 221 | 1  2.44 (0.93-6.44) | 0.071 |  | 1  2.16 (0.80–5.82) | 0.128 |
| Alcohol intake  <1 unit/week  1-4 units/week  ≥5 units/week | 219 | 1  2.22 (0.97-5.10)  3.48 (1.31-9.26) | 0.060  0.013 |  | 1  2.50 (1.05-5.97)  3.26 (1.20-8.89) | 0.039  0.021 |
| General health  *(Scoring 0–100, worst-best)* | 216 |  |  |  | 0.97 (0.95–0.99) | 0.011 |

*‘Limited HL’ was defined as reporting a limited level of general and/or electronic HL. ^a^Vegetables and fruit every day, fish 2/week, breakfast most days, pastries a few times/week.*

*NCP no chronic pain; CRP chronic regional pain; CWP chronic widespread pain.*
