# Supplementary material for: Health literacy in individuals with knee pain—a mixed methods study
Source: BMC Public Health. 2023 Aug 29;23:1656. doi: 10.1186/s12889-023-16585-9 (PMC10463821; doi:10.1186/s12889-023-16585-9)
Supplement: Supplementary file 5 — Additional file 5. [file 12889_2023_16585_MOESM5_ESM.docx]

**Additional file 5 – Interview guide**

**Main questions**

- How do you find information about health?
  - How often and why do you search for information about health?
  - How do you assess the credibility of the information regarding health issues?
  - How do you use the information you find/revive regarding your health?
  - How do you make decisions based on the information you receive?
  - Does it differ depending on where the information comes from?
- How do you find information about things/activities that can be good for your health?
  - How often and why do you search for information about related to healthy lifestyles?
- Where do you find information related to your knee pain?
  - How do you assess the credibility this information?
  - How do you use the information?
  - How do you make decisions based on the information you receive?
  - How do you act based on that information?
